# Titanium Elastic Nail (TEN) versus Reconstruction Plate Repair of Midshaft Clavicular Fractures: A Finite Element Study

**DOI:** 10.1371/journal.pone.0126131

**Published:** 2015-05-12

**Authors:** Langqing Zeng, Haifeng Wei, Yanjie Liu, Wen Zhang, Yao Pan, Wei Zhang, Changqing Zhang, Bingfang Zeng, Yunfeng Chen

**Affiliations:** 1 Department of Orthopaedics, Zhuhai People’s Hospital, Jinan University Affiliated Zhuhai Hospital, Guangdong, China; 2 Department of Orthopaedic Surgery, Shanghai Jiao Tong University Affiliated Sixth People’s Hospital, Shanghai Jiao Tong University, Shanghai, China; 3 Department of Orthopaedics, The First People’s Hospital Of Foshan, Guangdong, China; University of Umea, SWEDEN

## Abstract

**Background:**

The biomechanical characteristics of midshaft clavicular fractures treated with titanium elastic nail (TEN) is unclear. This study aimed to present a biomechanical finite element analysis of biomechanical characteristics involved in TEN fixation and reconstruction plate fixation for midshaft clavicular fractures.

**Methods:**

Finite element models of the intact clavicle and of midshaft clavicular fractures fixed with TEN and with a reconstruction plate were built. The distal clavicle displacement, peak stress, and stress distribution on the 3 finite element models were calculated under the axial compression and cantilever bending.

**Results:**

In both loading configurations, TEN generated the highest displacement of the distal clavicle, followed by the intact clavicle and the reconstruction plate. TEN showed higher peak bone and implant stresses, and is more likely to fail in both loading configurations compared with the reconstruction plate. TEN led to a stress distribution similar to that of the intact clavicle in both loading configurations, whereas the stress distribution with the reconstruction plate was nonphysiological in cantilever bending.

**Conclusions:**

TEN is generally preferable for treating simple displaced fractures of the midshaft clavicle, because it showed a stress distribution similar to the intact clavicle. However, TEN provides less stability, and excessive exercise of and weight bearing on the ipsilateral shoulder should be avoided in the early postoperative period. Fixation with a reconstruction plate was more stable but showed obvious stress shielding. Therefore, for patients with a demand for early return to activity, reconstruction plate fixation may be preferred.

## Introduction

The clavicle is a frequently fractured bone, accounting for 2.6–5% of all skeletal fractures [1].Approximately 80% of clavicle fractures involve the midshaft and more than half of these fractures are displaced [1,2]. To date, surgical treatment is the preferred approach for displaced midshaft clavicular fractures [3]. Open reduction with internal plate fixation and intramedullary fixation are two of the most commonly used surgical techniques for treating displaced midshaft clavicular fractures [4,5]. Plate fixation has been the more common method of operative treatment. However, intramedullary pinning provides an alternative method of fixation with improved functional outcomes and decreased nonunion rates in operatively treated patients [6].

Many recent studies have assessed the biomechanical characteristics of locking and nonlocking plate fixation of clavicular fractures [7–9]. A previous study showed that plate fixation provides more rigid stabilization than intramedullary fixation, and may provide a stronger construction for early rehabilitation protocols [10]. Another study evaluated the deformation mode, stress patterns, and peak stresses involved in superior and anteroinferior clavicle plate fixation by using the finite element (FE) method [11]. To date, no investigations have assessed the stress patterns (indicating how loads are transferred) and peak stresses (showing the likelihood of plate or bone failure) involved in intramedullary titanium elastic nail (TEN) fixation of the clavicle.

The purpose of this study was to compare the fixation of a midshaft fractured clavicle with TEN and with a reconstruction plate by using the FE method, in two clinically relevant loading configurations.

## Materials and Methods

### Three-dimensional FE model construction

A computed tomography (CT) data set of the left clavicle of a healthy 29-year-old male volunteer was obtained. The volunteer provided his written informed consent to participate in this study. The Ethics Committee of Shanghai Sixth People’s Hospital approve this study and the consent procedure. The subject was of 175 cm height and 77 kg weight, and had no previous injuries or diseases that could alter bone morphology. The scans were taken on a 16-slice CT equipment (Siemens Somatom Sensation 16, Forchheim, Germany) with 1-mm slice thickness and 0.453-mm in-plane resolution. The DICOM data were imported into Mimics 10.0(Materialise Leuven, Belgium), and a three-dimensional (3D) image was reconstructed and saved as an STL file. A 600 Hounsfield units (HU) value was used as an initial threshold value to separate the cortical and trabecular bones [12].

**Fig 1:**
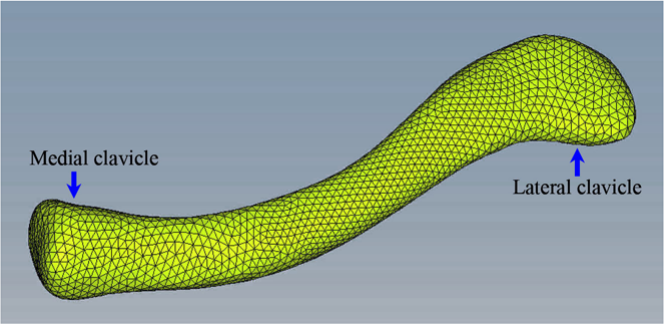
Superior view of the clavicle. Volume mesh generation resulting in 10-node tetrahedral elements.

**Fig 2:**
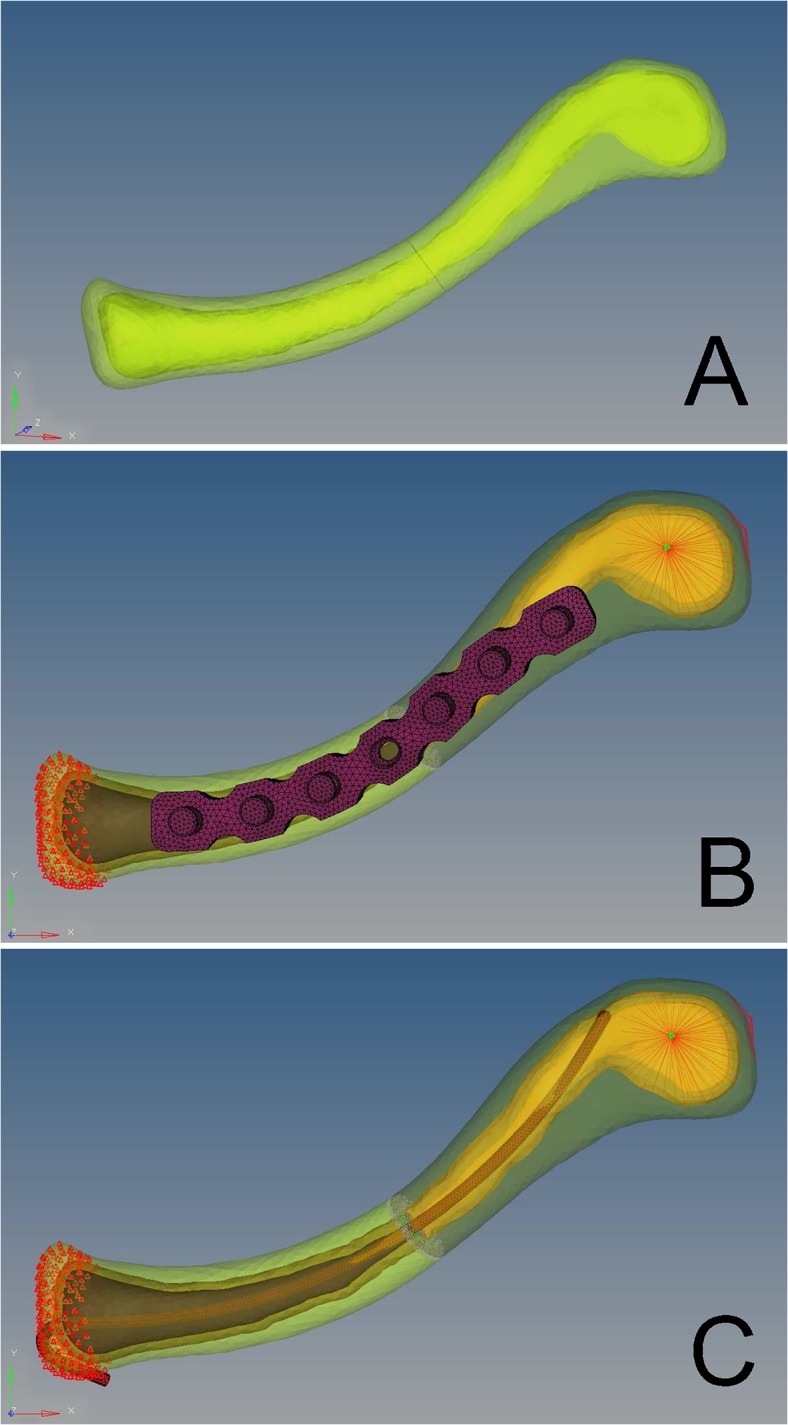
Finite element models. **(A)** intact clavicle. **(B)** midshaft clavicular fractures fixed with a reconstruction plate. **(C)** midshaft clavicular fractures fixed with titanium elastic nail.

The 3D geometry of the intact clavicle was imported into Geomagic 9.0(Geomagic, North Carolina,USA) for surface reconstruction. Next, the surface model was converted to 10-node tetrahedral FE meshes in Hypermesh 10.0 software (Altair, Troy, Michigan,USA)(Fig 1). The model was imported in Abaqus 6.9 software(Dassault Systems, MA, USA) for FE analysis. The finite element model of intact clavicle was given in Fig 2A. A simple midshaft fracture was simulated by creating a transverse gap of 1.0 mm width. The location of the fracture was in the middle of the FE model. A 7-hole, 3.5-mm reconstruction plate (Synthes, Switzerland) was modeled and fixed onto the superior contour of the clavicle with 6 bicortical locking screws, 3 screws on each side of the fracture. The screws were modelled as smooth, solid cylinder with a diameter of 3.5 mm. The plate was projected onto the bony surface to fit the superior contour of the clavicle (Fig 2B). For the TEN model, a TEN (Synthes, Switzerland) of 2.5 mm in diameter was selected [13],and modeled to fit the curved medullary canal of the clavicle.The TEN was inserted into the medullary cavity until the nail tip reached the cortical bone of the lateral clavicle (Fig 2C).All materials were considered linear-elastic and isotropic. The properties of the materials were taken from the literature [12,14]: cortical bone, elastic modulus 17 GPa, and Poisson's ratio 0.3; cancellous bone, elastic modulus 1.0 GPa, and Poisson's ratio 0.3; and titanium, elastic modulus 110 GPa, and Poisson's ratio 0.33. The number of nodes and elements of bone and implants are given in Table 1.

**Table 1:** Number of nodes and elements of bone and implants.

Model	Bone		Plate	TEN
	Cortical bone	Cancellous bone		
Node	4287	2844	6690	2762
Element	14,793	2840	23,782	8560

TEN, titanium elastic nail.

### Loads and boundary conditions

**Fig 3:**
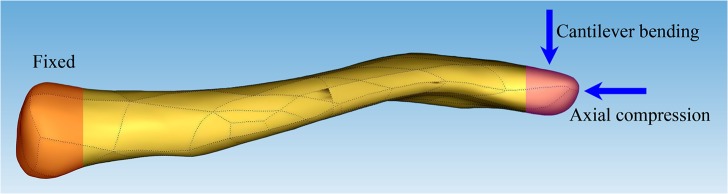
Diagram showing the loading directions of the 2 loading cases. The sternal side was held fixed in translation (orange). The external load was distributed over the distal side (purple), once for axial compression, and once for cantilever bending.

The contact interface was assigned between the TEN and bone,and between the proximal and distal clavicle.The interface between the screw head and plate was modeled tied interfaces.The contact between the screw and the bone was set as embedded elements, that is, the screws could not loosen or pull out. In this study, an axial compressive loading and a cantilever bending loading were investigated for each model according to Favre et al (Fig 3) [11]. In both loading cases, an arbitrary static total force of 250 N was equally distributed on the surface nodes situated at the 15-mm most distal part of the clavicle. The sternal nodes were constrained to remain fixed in translation [15]. In vivo rotations were possible at the sternoclavicular joint. However, rotations were restricted at the sternoclavicular joint in the model to avoid rigid body modes [15]. All other nodes were free to translate and rotate.

By analyzing the peak stresses for each model, and the average axial and vertical displacements of the 15-mm most distal part of the clavicle in each model, we can predict which implant provides greater stability. The normalized maximum von Mises stresses of the bone or implant was calculated accordingly as the peak stresses of the bone or implant divide the peak stresses of the intact clavicle during the corresponding loading conditions. The stress distributions involved in the TEN or plate fixation of the clavicle were analyzed, and by comparing with the distributions in the intact clavicle model, we can predict which implant would lead to a stress distribution similar to that in an intact clavicle.

### Model validation

To validate the model, the axial compressive rigidity of each reconstruction was normalized to the rigidity of the intact clavicle and then compared with previously published experimental data of the same models [11].The proximal side of the clavicle was held fixed, and the axial compressive rigidity was obtained by dividing the applied force with the average axial displacement of the 15-mm most distal part of the clavicle [11].

## Results

**Fig 4:**
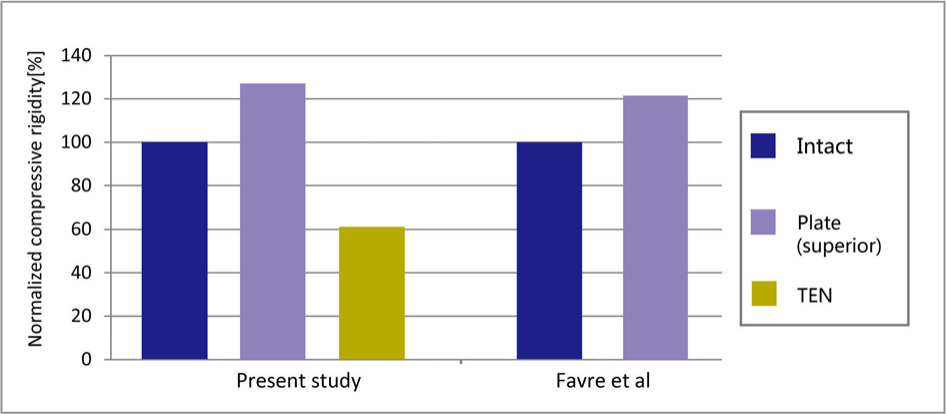
Construct rigidity in the intact clavicle and plate fixation (superior fracture fixation) for axial compression compared with the published experimental data [11]. The values obtained for the intact clavicle were set to 100% and served as a reference.The normalized compressive rigidity was 127.3%, 60.9% for the reconstruction plate model and TEN model, respectively.

**Fig 5:**
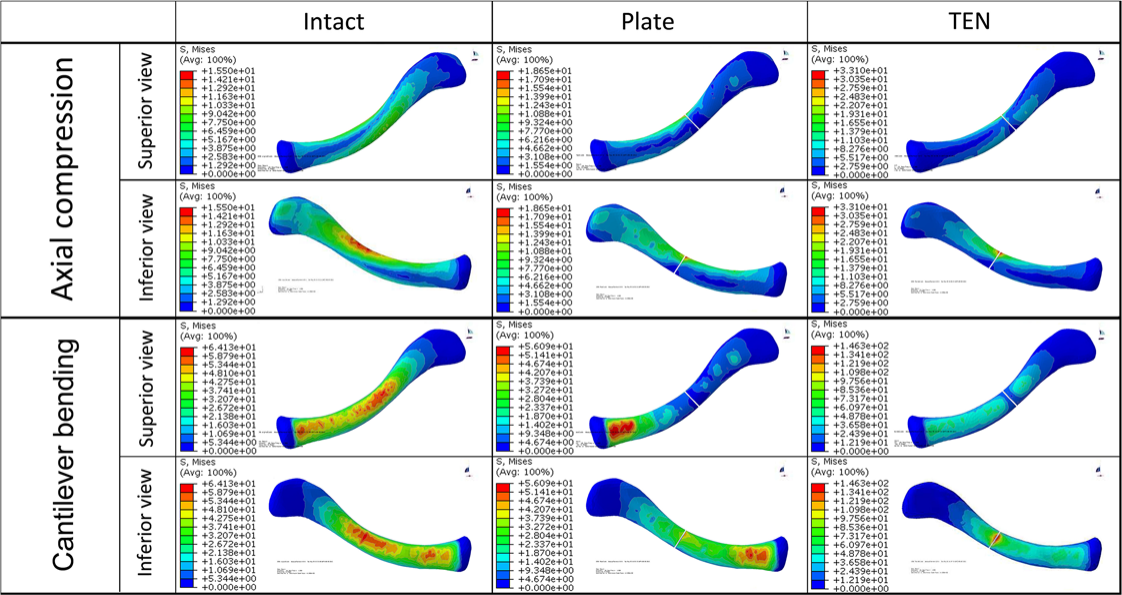
von Mises stress distribution in the bone of the three finite element models under both loading conditions.

**Fig 6:**
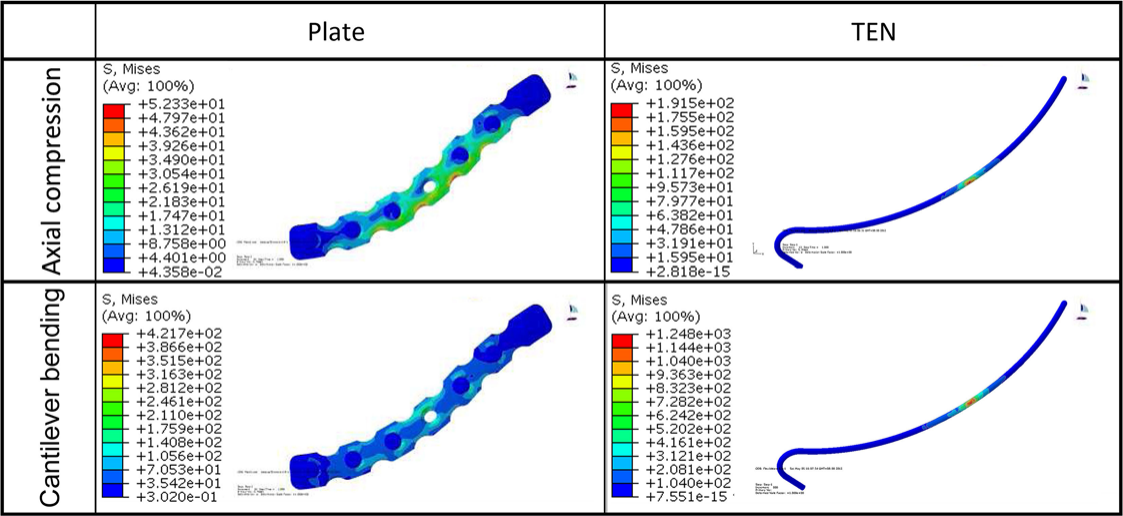
von Mises stress distribution in the implants for the two loading conditions. The superior view was chosen.

**Fig 7:**
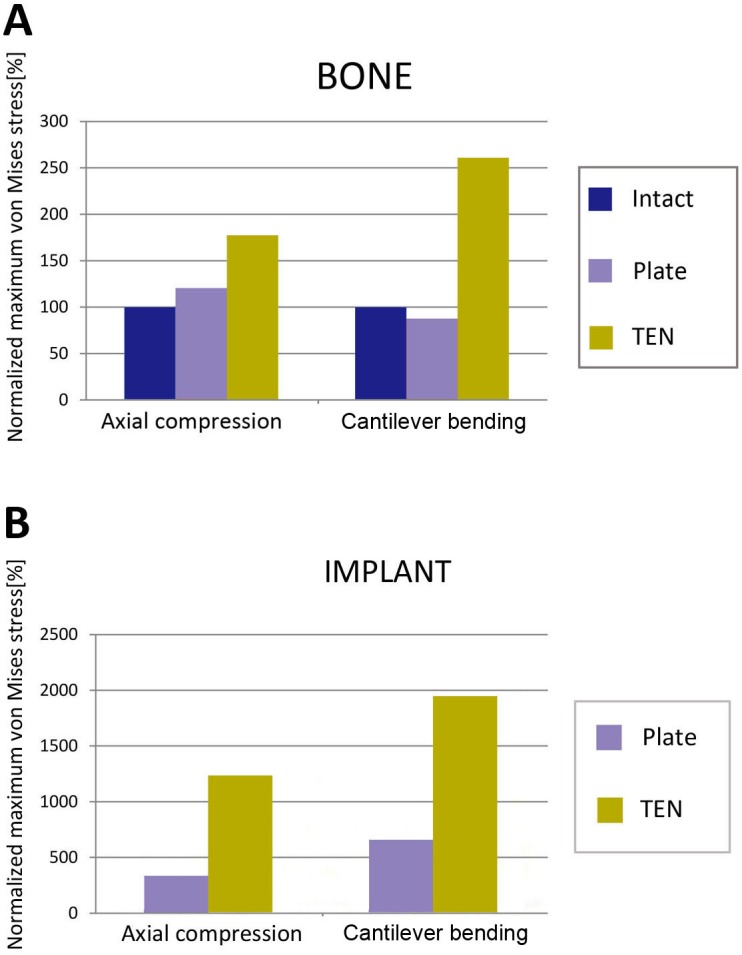
Peak von Mises stresses normalized to the stresses measured in the intact clavicle during the corresponding loading conditions for the bone (A) and the implant (B).

The normalized compressive rigidity of the reconstruction plate model was similar to the value measured experimentally (Fig 4).The average axial and vertical displacements of the 15-mm most distal portion of the clavicle are shown in Table 2. The von Mises stress distributions are shown for the clavicle (Fig 5) and for the implant (Fig 6). The peak stresses are given in Fig 7.

**Table 2:** Average axial and vertical displacements of the 15-mm most distal part of each model (mm).

Model	Intact	Plate	TEN
Axial compressive			
Axial displacement	0.14	0.11	0.23
Vertical displacement	0.26	0.17	0.36
Inferior bending			
Axial displacement	1.04	0.56	1.36
Vertical displacement	3.71	2.25	5.12

TEN, titanium elastic nail.

Both the average axial and vertical displacements of the 15-mm most distal portion of the clavicle under both loading conditions showed that TEN generated the highest displacement followed by the intact clavicle and the reconstruction plate. Results indicated that the plate is more stable than TEN for the treatment of simple displaced fractures of the midshaft clavicle.

For the intact clavicle, the peak stresses and the concentration of the stress were found in the middle of the clavicle as a result of the S-shape of the bone and the lowest flexural rigidity of this part [11,16]. The peak stresses of the clavicle were 15.50 MPa in the axial compression and 64.13 MPa in the cantilever bending. For the reconstruction plate fixation, the plate led to higher peak bone stresses (18.65 MPa) than in the intact clavicle in axial compressive loading (Fig 7A), with the stresses concentrated at the fracture site (Fig 5), and lower peak bone stresses (56.09 MPa) than in the intact clavicle in cantilever bending loading (Fig 7A), with the stresses concentrated around the most proximal locking screw (Fig 5). In both loading modes, the TEN fixation was associated with the highest peak bone stresses (33.10 MPa in axial compression, 146.30 MPa in cantilever bending) (Fig 7A), with the stresses being concentrated at the fracture site (Fig 5). Additionally, TEN was associated with higher peak implant stresses (191.50 MPa in axial compression, 1248.00 MPa in cantilever bending) and showed more significant stress concentration at the site of fracture than the reconstruction plate in both loading conditions (52.33 MPa in axial compression, 421.70 MPa in cantilever bending) (Figs 6 and 7B). The results indicated that TEN fixation is more likely to fail in both loading modes compared with reconstruction plate fixation.

For the stress distribution on the clavicle, both the TEN model and the reconstruction plate model were similar to the intact clavicle in axial compressive loading, with the stresses concentrated at the middle of clavicle (including the fracture site) and transmitted to the sternal and acromial ends of clavicle (Fig 5). The TEN model showed a similar stress distribution to that in the intact clavicle in cantilever bending, with the stresses being concentrated at the middle of clavicle, followed by the sternal end of the clavicle, and dispersed at the acromial end of the clavicle (Fig 5). However, the stress distribution in the reconstruction plate model was different from that in the intact clavicle, with the stresses obviously concentrated around the most proximal locking screw (the proximal part of the clavicle) and transmitted to the acromial end of the clavicle (Fig 5). These results indicated that reconstruction plate fixation has an obvious stress shielding effect in cantilever bending.

## Discussion

With the increasing interest in the use of TEN for treating midshaft clavicular fractures, understanding the mechanics of TEN can improve treatment. This study compares the biomechanical properties of TEN and reconstruction plate repair of midshaft clavicular fractures by using the FE method in both the axial compressive and cantilever bending loading modes.

According to our results, TEN fixation provides less stability and is more likely to fail than reconstruction plate fixation for simple displaced midshaft clavicle fractures, on the basis of the larger displacement of the 15-mm most distal part of the clavicle and the greater peak bone and implant stresses in both loading modes. However, the TEN fixation model induced stress distributions similar to those in the intact clavicle in both loading configurations, whereas the stress distribution with the reconstruction plate fixation model was nonphysiological in the cantilever bending configuration. Favre et al [11] evaluated the biomechanical properties of superior and anteroinferior clavicle plate fixation by using the FE method. The authors reported that anteroinferior clavicle plate fixation led to a deformation mode similar to that in the intact clavicle in both loading configurations, whereas the deformation mode with superior clavicle plate fixation was nonphysiological.

To date, open reduction and internal fixation with reconstruction plates and elastic stable intramedullary nailing with TEN are two of the most commonly used surgical techniques for treating displaced midshaft clavicular fractures [4,5,13,17]. Plate fixation and intramedullary fixation have their own advantages and disadvantages. Plate fixation can provide more rigid stabilization than intramedullary pin fixation does, and may help facilitate early mobilization and offer a superior construct for highly comminuted fractures where the bridge plating technique can be implemented [10,18–20]. However, this technique may require large incisions and extensive exposure, which could cause complications such as infection, scarring, and refracture after the removal of the plate [21,22]. Intramedullary fixation provides an alternative and less invasive technique for the treatment of displaced midshaft clavicular fractures; it has the advantages of obtaining relatively stable fixation that allows axial compression, and preserving the soft tissue envelope, the periosteum and the vascular integrity of the fracture site, which enhances healing [20,23]. Chen et al [13] reported that TEN fixation allows for earlier relief of shoulder pain and a more cosmetically satisfactory appearance than plate fixation. In addition, the infection rates may be decreased and fracture callus formation enhanced [23].However, the main complications of intramedullary fixation are hardware migration, skin irritation, and the requirement for routine removal of the intramedullary pin after fracture healing [13,17,24]. Intramedullary pins also provide less rotational, bending, and length stability compared with plate fixation techniques [10,18,25]. Smith et al [26] compared a six-hole pre-contoured locking plate to a 4.0 mm intramedullary clavicle fixation device. They found that intramedullary devices and plate devices provide similar repair strength for middle-third clavicle fractures in response to bending load to failure[26]. However, the intramedullary device removal group to be significantly stronger than the plate removal group for testing of the hardware removal groups to simulate device removal after fracture union[26]. Therefore, there is no standard treatment for treating displaced midshaft clavicular fractures. A systematic review performed by Houwert et a [4] showed no difference in functional outcome or complications after plate fixation or intramedullary fixation for displaced midshaft clavicle fractures.

On the basis of our results, TEN is more suitable for the treatment of simple displaced midshaft clavicle fractures because it showed similar stress distributions to those observed in the intact clavicle [27]. Additionally, TEN fixation has the advantages of being less invasive and providing higher patient satisfaction and a more cosmetically satisfactory appearance compared with plate fixation [13]. However, it should be noted that TEN fixation had greater peak bone and implant stresses in the same loading conditions compared with plate fixation. This indicates that excessive exercise of the ipsilateral shoulder should be avoided in the early postoperative period, to prevent excessive bone and implant stresses, implant failure, and interfering with bone union. Therefore, in the study by Chen et al [28], the affected shoulder was allowed to receive passive non-weight-bearing exercises immediately after surgery, which continued for 2 weeks, and the range of shoulder abduction was gradually increased but kept within 90°during the first 3 weeks after surgery. Conversely, plate fixation is more suitable for comminuted midshaft clavicle fractures or for patients with a demand for early return to activity, on the basis of its more rigid stabilization, helping maintain the length of clavicle and facilitate early mobilization [10,27].

This study has some limitations that should be considered. First, the geometry of the FE model was based on a single clavicle from a young male volunteer. Variations in clavicle geometry can be important but were not considered in the present study [16]. However, young male persons have a higher risk for clavicle fractures [1,29]. Second, we built an oversimplified midshaft clavicle fracture model, although we are aware that the types of clavicle fracture are much more complex in the clinical setting. Third, an arbitrary static total force of 250 N for both the axial compression and cantilever bending loads was set based on Favre et al [11], and the effect of the surrounding soft tissues on the mechanical stability of the construct was not evaluated. In studies involving clinical observation, axial compression, and cantilever bending were both suggested to occur in vivo [29,30]. However, in vivo loads acting on the clavicle during the activities of daily living are an intricate combination of muscular and external forces that remain unknown to date [15,31]. Fourth, the FE models were validated by the date of previously study [11], instead of a biomechanical test result.Finally, the mechanical behavior may be influenced by the diameter of TEN. A TEN diameter of 2.5 mm was chosen to reconstruct the model for analysis because the diameter of the medullary cavity of the clavicle ranges from 2.8 to 3.0 mm in most patients, and a 2.5-mm TEN is most commonly used [28].Despite these limitations, the present study provides a comparison of the mechanical behavior of TEN fixation and reconstruction plate fixation for midshaft clavicular fractures, using the same conditions and assumptions for analysis. Randomized controlled trials with long-term follow-up are required to corroborate the results of this study.

## Conclusion

TEN is suitable for treating simple displaced fractures of the midshaft clavicle because it showed a stress distribution similar to the intact clavicle under both loading configurations analyzed in this study. However, TEN fixation provides less stability compared with plate fixation, and therefore excessive exercise of and weight bearing on the ipsilateral shoulder should be avoided in the early postoperative period. Reconstruction plate fixation for midshaft clavicular fractures showed greater stability, but had obvious stress shielding. Therefore, for patients with a demand for early return to activity, reconstruction plate fixation may be preferred.
